# Team-based learning (TBL): a community of practice

**DOI:** 10.1186/s12909-019-1795-4

**Published:** 2019-10-15

**Authors:** Annette Burgess, Inam Haq, Jane Bleasel, Chris Roberts, Roger Garsia, Nicholas Randal, Craig Mellis

**Affiliations:** 1 Faculty of Medicine and Health, University of Sydney School of Medicine, Education Office, University of Sydney, Sydney, Australia; 2 Faculty of Medicine and Health, University of Sydney School of Medicine, Sydney Health Education Research Network, University of Sydney, Sydney, Australia; 3 Faculty of Medicine and Health, University of Sydney, Sydney, Australia; 4 Faculty of Medicine and Health ,University of Sydney School of Medicine, Northern Clinical School, University of Sydney, Sydney, Australia; 5 Faculty of Medicine and Health ,University of Sydney School of Medicine, Central Clinical School, University of Sydney, Sydney, Australia

**Keywords:** Team-based learning, Communities of practice, Flipped classroom

## Abstract

**Background:**

Rapid changes in medical practice have a large impact on the demands faced by educators in preparing students for future participation in a multifaceted healthcare workforce. Competencies required by today’s medical graduates encompass the ability to effectively collaborate, communicate and problem solve. The learning needs of medical students have also changed over time. Today’s medical students are highly interconnected, enjoying teamwork and collaborative practice, and desire continuous, explicit feedback. They want structured learning activities, with clear expectations, and enjoy a sense of accomplishment on their achievements. The conflation of these issues has seen many medical schools adopt the model of Team-based learning (TBL). Using the conceptual framework of communities of practice, we sought to qualitatively explore students’ and teachers’ experience of TBL in Year 1 of a graduate entry medical program.

**Methods:**

Convenience sampling was used to select 169/350 (48%) Year 1 students who completed three TBL sessions. Each TBL session was facilitated by three senior clinicians. Following participation in the TBLs, students were invited to attend focus groups, and all facilitators (*n* = 9) were invited to attend interviews. A coding framework was developed to code the entire dataset, using the theoretical lens of communities of practice.

**Results:**

34/169 (20%) of students attended focus groups. Three facilitators (3/9, 33%) were interviewed. Students and facilitators felt the structure and organisation of TBL made students accountable for their learning and team contributions. The combined expertise and clinical experience of facilitators, with immediate feedback helped groups to work both independently and collaboratively. Facilitators found working with their peers in the TBLs to be a rewarding experience.

**Conclusions:**

The community of practice found in the TBL classes, provided an enriching and rewarding learning environment that motivated students to build on their basic knowledge and apply what had been learnt. The interactions of experienced, senior clinicians as facilitators, sharing their expertise within a clinical context, prompted effective student engagement in learning and understanding. Our change in curriculum design and pedagogy will assist in preparing medical students for demands of the increasingly complex healthcare systems in which they will work.

## Background

Rapid changes in medical practice have a large impact on the demands faced by educators in preparing students for future participation in a multifaceted healthcare workforce. The practice of medicine increasingly requires multi-professional team-work in order to provide the best patient outcomes [1]. Competencies required by medical graduates encompass the ability to effectively collaborate, communicate and problem solve. Graduates must be trained as lifelong learners, capable of accessing, assessing and synthesising a wealth of information relevant to health care [1]. For at least four decades many medical schools have relied on the learning environment provided within a Problem Based Learning (PBL) approach, in which tutors facilitate small groups so as to allow self-directed learning and experiential learning activity. However, larger class sizes and budget constraints have seen the requirement for new models of small group teaching that promote collaborative learning [2].

The learning needs of medical students are also thought to have changed over time. Today’s medical students are highly interconnected, enjoying teamwork and collaborative practice, and the use of social media for learning [3]. They are also reported to have a unique outlook on assessment, desiring continuous, explicit feedback. They want structured learning activities, with clear expectations, and enjoy a sense of accomplishment on their achievements. In addition to modifications in teaching methods, educators have embraced technological advancement in the delivery of medical education. Adopting blended learning models has the potential to enhance student engagement both inside and outside of the class room [4]. In particular, the ‘flipped’ classroom approach has the capacity to maintain the collaborative nature of learning within large class structures [5], and is being increasingly adopted in health professional education [6]. The conflation of these issues has seen many medical schools adopt the model of Team-based learning in place of Problem-based learning [7, 8].

Within medical education concerns have been raised with the larger class size approach in TBL, the use of the flipped classroom approach; the reduction in group level facilitation in favour of class level facilitation; and the competitive nature of the readiness assurance testing. These concerns suggest that the learning environment in TBL isn’t conducive to co-operative or collaborative learning. However, there is very little literature reported addressing this. An opportunity to fill this gap arose with the introduction of TBL in Year 1 of a hybrid PBL graduate entry medical program in a large research intensive University, as a small group, blended teaching method in place of PBL [9]. In the study reported in this paper, we were interested to qualitatively explore students’ and teachers’ experience of TBL from a social and collaborative learning perspective.

### Conceptual framework

Sociocultural learning theories assist our understanding of how students learn from each other, and how teachers construct their learning environments. It is thought that learning environments influence students’ learning process through regular social interactions with peers, teachers and experts. Teachers have the capacity to create learning environments that maximise learning ability by supporting collaboration, discussion and feedback. The theoretical concept of communities of practice is often utilised in medical education literature [10]. First described by Lave & Wenger, there are three key structural elements to the communities of practice [11]:

1. *Joint enterprise:* a shared domain of interest and a shared desire for proficiency in a subject
2. *Mutual engagement:* joint activities that promote collaboration and development of learning relationships
3. *Shared repertoire:* promotion of a shared language, resources, concepts, experiences and tools used and develop through interactions

By understanding the factors that assist in the development of a community of practice within the TBL structure and setting, we sought to address the research gap by qualitatively exploring students’ and teachers’ experience and views of TBL in Year 1 of a graduate entry medical program. Our specific research question was, “What are the students’ and teachers’ experience and views of TBL in Year 1 of a graduate entry program, as considered through the conceptual lens of communities of practice?”

## Methods

### Study context

Although the context of the study has been previously described [9], the data collected and analysed in this current study has not been previously used, and the research question for this study is unique. In 2016, at the time of this study, the Sydney Medical Program (SMP) offered a hybrid PBL based curriculum within its 4 year graduate entry medical program. Students attended PBL classes twice per week, for 1.5 h each class.

### Sampling and participants

This study was carried out in 2016, when there was a cohort of 350 Year 1 medical students enrolled in the University of Sydney medical program. Convenience sampling was used to select 169/350 (48%) of these Year 1 students, who were required to complete three TBL sessions during one of the following teaching blocks: Musculoskeletal, Respiratory or Cardiovascular. Students were assigned to one of three TBL ‘classes’: Musculoskeletal (*n* = 56), Respiratory (*n* = 59) or Cardiovascular (*n* = 54). Each student within this sample completed a total of three TBLs within their class. Within each of these classes, students were assigned to their permanent TBL teams, consisting of five or six students per team. Students were assigned to their teams based on whether they had a science or non-science background, and on gender, so that each team had a diverse mix of students.

### Structure of team-based learning

The TBL sessions were held once per week for 2 hours. Nine senior academic clinicians participated as facilitators: three Rheumatologists, three Respiratory physicians, and three Cardiologists. Facilitators consistently attended every TBL in their specialty, that is facilitator teams were consistent. TBL methods were followed as outlined in Table 1. Out of class, we provided pre-class preparation by way of essential online readings and/or pre-recorded lectures. In class we delivered the individual readiness assurance test (IRAT), consisting of 10 multiple choice questions, with one single best answer. This was followed by the very same test being delivered as a team readiness assurance test (TRAT), followed by immediate feedback led by the content experts. Students then moved on to the clinical case-based problem-solving activities, consisting of approximately five problems that were based around a patient case.

**Table 1:** TBL structure

*Pre-class reading (1–2 h)*	Prior to class, students were allocated compulsory readings and/or pre-recorded lectures.
*In-class schedule (2 h)*	10 mins: Individual Readiness Assurance Test (IRAT); 20 mins: Team Readiness Assurance Test (TRAT); 20 min: Immediate feedback from the facilitators; 60 min: Clinical problem solving activities; 10 mins: Close.

### Data collection and analysis

At the completion of each block of teaching, all students who participated in the TBLs (*n* = 169) were invited to attend focus groups. Additionally, all facilitators were invited to attend individual interviews. Semi-structured question guides developed from communities of practice literature, and from discussion with the all authors were used to lead the focus groups and interviews. The focus groups and interviews were conducted by the first author, an experienced medical education researcher, trained in the facilitation of focus groups and interviews. Focus groups and interviews were recorded and transcribed verbatim. Thematic analysis was undertaken using Framework Analysis [12]. This was conducted by the first author on a sample of the data, with the aim to identify recurrent themes and subthemes in the dataset and inform the development of a coding framework. Following a discussion with all authors, a coding framework was developed to code the entire dataset through the theoretical lens of communities of practice.

### Ethics approval

Ethics approval was gained from the University of Sydney Human Research Ethics Committee. Approval project number: 2016/136.

## Results

In total 34/169 (20%) of students who participated in the TBLs attended one of five focus groups. Two focus groups were held at the end of the Rheumatology block, two at the end of the Respiratory block, and one at the end of the Cardiology block. Of the student focus group participants, 19 were male, and 15 were female. Three facilitators (3/9, 33%) were interviewed, including two rheumatologists and one respiratory physician.

All data from the focus groups and interviews are presented using the conceptual framework of the communities of practice. The theme of ‘joint enterprise’ is illustrated in Table 2. Students and facilitators found that the specified pre-reading and pre-recorded lectures ensured that students came to class with sufficient requisite knowledge as a mechanism to increase their willingness to integrate and apply this information, ultimately increasing their engagement. In class, the combined expertise and clinical experience of facilitators, with immediate feedback helped groups to work both independently and collaboratively. Facilitators found working with their peers in the TBLs to be a rewarding experience. The theme of ‘mutual engagement’ is illustrated in Table 3. Students and staff found the TRAT, and the use of small groups promoted collaboration and teamwork, but also gave individual students equal opportunity to contribute to their team. Facilitators felt the structure and organisation of TBL made students accountable for their learning and team contributions, and also brought about practical efficiencies in learning and teaching. The theme of ‘shared repertoire’ is illustrated in Table 4. Students and facilitators agreed that the use of authentic clinical problems in TBL provided an opportunity to improve student understanding by encouraging self-reflection, and the means to identify knowledge gaps, and build on prior knowledge.

**Table 2:** Participants’ responses regarding perceptions of their experiences that related to “Joint enterprise”

JOINT ENTERPRISE: shared domain of interest and a desire for proficiency in a subject	
**Co-teaching** Facilitators found it beneficial and enjoyable to teach alongside other experts in their field	*“Positive experience for facilitators. Working with other expert teachers in my area and therefore able to bounce off ideas. It was a positive experience for the facilitators”. (facilitator)* *“Working with other “experts” in a very collegial atmosphere is also very rewarding, and being able to interact with the students as we wander around the table, being available for questions”. (facilitator)*
**Content experts as facilitators** Staff felt TBL improved the quality of teaching by providing content experts as facilitators. Staff enjoyed sharing their expertise in subjects	*“The team-based learning overcomes inequity for student groups by having a small number of well trained content experts rather than having multiple tutors….you just can’t provide enough high quality tutors to do PBL”. (facilitator)* *“I enjoyed going through the MCQs & elaborating on the correct answers ..ie a short didactic/highly focused ‘mini lecture’” (facilitator)*
**Immediate feedback** Students felt that immediate feedback from a content expert helped increase the independence of teams, and improved the continuity while working through their problem solving activities. Teachers felt the immediate feedback encouraged a desire for subject proficiency among students.	*“I felt like we were a lot – a lot more independent in TBL than in PBL… it’s like babysitting in PBL, whereas TBL we could, kind of, take on the clinical case ourselves and then experts come in to clarify points, then leave again… we control how – how fast we go”. (student)* *“After they have done the individual and the team test, the facilitator goes through the answers to clarify any misconceptions. That’s actually really fantastic for the students because it produces some competition, it encourages them that if they are not doing as well as the other teams, for the next session, to look more closely at the information that is set for learning”. (facilitator)*
**Flipped classroom model, same preparation for every student** Students felt that having the same preparation requirements for team members in TBL, rather than having different individual preparation requirements (as is the case in PBL), engaged team members to work effectively together on a clinical problem.	*“Everyone on is on the same page because you’ve all watched the same video (pre-recorded lecture) coming in and then afterwards you’ve all got the same questions and you can work through the questions and what your thought process was and think about it, whereas, if you do research on “x” and say, great, now I know about “x” but now I’ve got to still learn everybody else’s learning topics as well”. (student)* *“Coming to TBL, it’s kind of that expectation that your four other members in your group have already done it (prepared) so it’s on you to also do it as well. And that’s qualified by the fact that there are questions, so you have to have knowledge, you can’t just breeze through like PBL”. (student)*

**Table 3:** Participants’ responses regarding perceptions of their experiences that related to “Mutual engagement”

MUTUAL ENGAGEMENT: joint activities that promote collaboration and development of learning relationships	
**Structure and steps in TBL promoted student preparation and collaboration** The specific in-class steps of TBL, including the pre-reading, the individual and team test and a series of problem solving activities encouraged students to work together. Students were made accountable for their learning both individually and as a group, thus promoting team development. Facilitators felt the structure of TBL helped ensure that students come to class prepared to engage in joint activities.	*“Doing the test you might go through by yourself, not get all the questions right, you’ve got other people’s brains to bounce off, it’s more the way that it is – because both of them are guided in a manner but I feel like TBL’s much more guided around, knowledge and learning where I found PBL was very guided around time.” (student)* *“TBL is very structured. There is pre-reading that is compulsory for the students, and they do it because they are assessed as soon as they come into the room. They do an individual test, and then the team does the same test. There is a lot of accountability on the students, a lot of competitiveness, so they are really doing a proper flipped class room, they actually do come along prepared”. (facilitator)*
**All students in one large class-room space** Staff and students felt having all groups of students in one large room facilitated intra and inter-team collaboration, creating a relaxed, safe environment and collegial atmosphere for student learning.	*“I think the experiences I’ve had about why TBL works so well is the organization – you’ve got to have the right space for students to work in small groups, but also mix with each other…..they have an opportunity to share in a safe space, which is fantastic”. (facilitator)* *“…..the fact that it was in this big room, we were collaborating still, the competition wasn’t too serious, but it added an element of pressure or motivation to the group dynamic”. (student)* *“..if you’re struggling with something like drawing the mechanism, it’s just not working, you look immediately to your left and you have a good example of how you could do it and in PBL - you’re locked in a room in this big corridor it’s completely isolating. We lean across and discuss, and the tutors would ask people their different thoughts, there was that sharing as well….like cross group learning”. (student)*
**Small group size of TBL teams** The small size of the TBL groups (five students per team) compared to PBL groups (10 students per team) motivated students to prepare for the class and contribute to discussions.	*“PBL is just excruciating that where same people talk every week, the same people are quiet every week and there’s nothing that changes that, but I think in TBL even if someone’s a person who’s inclined to be quiet and inclined to, kind of, shy away from their opinion, just the environment of the room makes people change … because it’s much more collaborative, it’s much more friendly ..much more stimulating”.* (student)
**Use of testing prompted students to prepare** In TBL, individual accountability and effective teamwork was fostered by the individual and teams tests. Testing at the beginning of class was an important influence in ensuring students came to class prepared. The team test in TBL promoted friendly competition between teams, and active discussion within teams.	*“I think there was the competitive edge, as bad as it sounds, no one wants to be the loser. There’s the whole thing if you didn’t watch the lectures and people look at you.. get the vibe. You turn around to your friend and joking be like, ‘yeah, you better prepare for next week mate, we’ve got to win’”. (student)* *“People had to explain what they thought was right or wrong, and I think this is a really good way of getting people to work together as they will do in the future in the medical profession”. (student)*

**Table 4:** Participants’ responses regarding perceptions of their experiences that related to “Shared repertoire”

*SHARED REPERTOIRE: promotion of a shared language, resources, concepts, experiences and tools used and develop through interactions*	
**The team test prompted professional skills in critical reflection and collaboration** In TBL, students felt the individual test and team test at the beginning of class reinforced key concepts of the topic, increased confidence, and enabled critical reflection. The team test supported opportunities to explore and view knowledge in different ways. With an emphasis on active learner involvement, where students were tackling problems together, the students’ learning and reflection process was enhanced.	*“The good thing about the tests, both doing it individually and as a group, is, I found it a boost of confidence -- because I would actually remember this stuff and then, learning how to back yourself when you’re maybe unsure that you actually know information and presenting it to people and being able to be, like, no, this is right because of this….. that’s a pretty important step I think…. speaking up more…backing yourself”. (student)* *“(In the team test) every single person changed an answer, so we had a couple of people who were really, really strong, and they changed their answer because of the group discussion. ….it’s like legitimate cooperation, with an outcome of, I actually learnt something from these people I was collaborating with…like what a miracle”. (student)*
**Development of clinical reasoning skills** Facilitators felt students were able to seek appropriate information from peers and from the facilitators in order to solve authentic clinical problems. Students felt clinical reasoning was assisted by provision of clarification from the expert tutors, particularly around the depth and breadth of basic science relevant to a clinical problem.	*“The expert advice they get from the facilitators is what they need, which is an advantage over PBL. It is really good to see students work through an authentic clinical problem and seeing them ask the right questions around clinical reasoning or understanding scientific principles and being able to give them the up to date, evidence based answers”. (facilitator)* *“I think the other good thing about having the experts is if we want to question something or challenge something, there’s space to do that … The point of the session is to address an illness, a disease and you need clinical reasoning for that – you need a clinician here”. (student)*
**Problem solving activities** Problem solving activities promoted collaboration among small teams and the TBL class. Students felt they used the steps and tools of TBL to produce an end product during each class (mechanistic flow chart).	*“The problem solving activities that we did through the TBL sessions were quite a good way to investigate the topic. The way that our groups worked was that we followed through a series of questions to make a chart in the end about the risk factors and the signs and symptoms of the diseases and then a great element was to be able to compare our groups chart with the other groups’ charts, and see maybe a different method of preparing the chart, or see what we missed in our chart and other groups had”. (student)*

## Discussion

We sought to qualitatively explore students’ and teachers’ perceptions of TBL during Year 1 of the Sydney Medical Program, using the theoretical framework of ‘communities of practice’. Within this framework, knowledge is developed as a social, rather than individual feature, which hinges on the concept of “distributed cognition”, where students are dependent upon the knowledge of their peers and resources. In our TBL model, learning involved a process of collaboration within teams, and also between teams as a larger student body, where basic science and medical knowledge and skills were socially constructed. While there is some overlap between themes, the students’ and teachers’ joint enterprise, mutual engagement, and shared repertoire in the TBL classes facilitated learning and enriched the class environment.

### Joint enterprise

Joint enterprise refers to a shared domain of interest, and a shared desire for proficiency in a subject [11]. Co-teaching in TBL, with meaningful teaching-learning processes to amalgamate theoretical knowledge with clinical application, was appealing to both teachers and learners.

Teachers described the positive experience of *“working with other experts in a collegial atmosphere”* as “*rewarding”* and *“positive*”. The guidance and feedback they provided to students as co-teachers, formed an integral part of the class process. In line with our findings, evidence suggests that co-teaching is effective in generating student interest, engagement, knowledge acquisition and retention [13]. Students reported the immediate feedback, and relevant clinical context provided by the specialist clinicians, helped their learning. The facilitators *“enjoy(ed) going through the MCQs and elaborating on the correct answers”*. They shared information and experience from their specialised areas, giving students more motivation and purpose to master that subject. Students felt having experts as teachers shaped the quality of their learning.

Students and teachers alike valued the ‘flipped classroom’ format of TBL. Students felt the online pre-reading and recorded lectures enhanced their engagement in the subsequent TBL classes, particularly in the small-group work. Order and commitment were gained by students having the same preparation requirements. The benefits of the flipped classroom model are reported as being the use of more complex cases, with an increased opportunity for clinically relevant teaching; and enhanced teamwork, with students building on their peers’ knowledge and skills (Chen et al., 2017).

### Mutual engagement

Mutual engagement refers to joint activities that promote collaboration and development of learning relationships [11]. The specific steps and structure of the TBL process (preparation, IRAT, TRAT, problem solving activities, feedback) helped to engage students. This is in line with literature suggesting that in a community of practice, students move beyond active learning as individuals by participating in structured, collaborative learning activities that are engaging, interactive and relevant [14].

A clear strength of the TBL was having multiple groups in the one room, and having small individual teams of five to six students. Inter- and intra-group relationships were developed with guidance from facilitators. Students benefited from this participation, largely through the power of interaction, with the development of friendly competition and camaraderie. Active learning opportunities that engage participants have the potential to assist in the development of a deeper understanding of knowledge and increase knowledge retention [15].

Working together on tests and problem solving, in small groups of five or six students, provoked ongoing dialogue between group members, to gain consensus and build on each other’s individual knowledge. Students were dependent upon each other for their knowledge, which also fostered this collaborative learning. Vygotsky’s Zone of Proximal Development (ZDP) indicates the breadth and depth of learning possible by a student when provided with instruction [16].

### Shared repertoire

Shared repertoire refers to the collective acquisition of shared language, resources, concepts, experiences and tools used and developed through interactions [11]. Throughout the course of the TBL sessions, students became familiar with the TBL teaching methods and resources. Self-directed learning within groups was still possible through established routines of pre-class reading, in class tests, receipt of feedback and completion of problem-solving activities. Students’ knowledge base was developed through their shared practices, such as the method used to construct the team’s pathophysiological flow chart.

The team test supported opportunities for students to explore and view knowledge in different ways, promoting self-reflection. Both the team test and clinical problem-solving activities made students reliant upon their collective experiences and understanding of knowledge, prompting critical reflection. Critical reflection is recognised as a method of analysing information to prepare for practice [17]. When an emphasis is placed on active learner involvement, students are encouraged to tackle problems together, in order to enhance the learning and reflection process [18]. Additionally, placing an emphasis on the basic sciences within a clinical context, has been shown to heighten interest and curiosity in the learner [8, 19]. In TBL, students reported that construction of their knowledge was assisted with provision of a relevant and authentic clinical context from experienced clinicians.

#### Study limitations

The results of this study are based only on perceptions of students and facilitators. Students and staff voluntarily took part in the focus groups and interviews, which may have biased our results. Their views may or may not be representative of the wider student or staff population, or applicable to other universities**.**

## Conclusion

The socialisation, teaching and learning methods encouraged and entrenched in the strategies of team-based learning have been described using the communities of practice theoretical framework. The community of practice found in the TBL classes, and enhanced through the structure of TBL, provided an enriching and rewarding learning environment that motivated students to build on their basic knowledge and apply what had been learnt. Facilitators enjoyed the experience of helping students to construct their knowledge within the TBL framework, and social practice was developed. The interactions of experienced, senior clinicians as facilitators, sharing their expertise within a clinical context, prompted effective student engagement in learning and understanding. Our change in curriculum design and pedagogy will assist in preparing medical students for demands of the increasingly complex healthcare systems in which they will work. Future research investigations will utilise an ethnographic study design to provide a rich understanding of how students think and learn together within the TBL community of practice.

## Data Availability

The datasets generated and/or analysed during the current study are not publicly available due the confidentiality agreements approved by the Human Research Ethics Committee, University of Sydney, but are available from the corresponding author on reasonable request.

## Abbreviations

IRAT: Individual Readiness Assurance Test
PBL: Problem based learning
SMP: Sydney Medical Program
SMS: Sydney Medical School
TBL: Team-based learning
TRAT: Team Readiness Assurance Test
